# The Acute Effects of Low-Dose TNF-**α** on Glucose Metabolism and **β**-Cell Function in Humans

**DOI:** 10.1155/2014/295478

**Published:** 2014-02-16

**Authors:** Tobias Ibfelt, Christian P. Fischer, Peter Plomgaard, Gerrit van Hall, Bente Klarlund Pedersen

**Affiliations:** ^1^Department of Infectious Diseases and CMRC, The Centre of Inflammation and Metabolism (CIM), Rigshospitalet, Faculty of Health Sciences, University of Copenhagen, 7641 Blegdamsvej 9, 2100 Copenhagen, Denmark; ^2^Copenhagen Muscle Research Centre, Rigshospitalet, 2100 Copenhagen, Denmark; ^3^Department of Biomedical Sciences, University of Copenhagen, 2100 Copenhagen, Denmark

## Abstract

Type 2 diabetes is characterized by increased insulin resistance and impaired insulin secretion. Type 2 diabetes is also associated with low-grade inflammation and increased levels of proinflammatory cytokines such as TNF-**α**. TNF-**α** has been shown to impair peripheral insulin signaling *in vitro *and *in vivo*. However, it is unclear whether TNF-**α** may also affect endogenous glucose production (EGP) during fasting and glucose-stimulated insulin secretion (GSIS) *in vivo*. We hypothesized that low-dose TNF-**α** would increase EGP and attenuate GSIS. Recombinant human TNF-**α** or placebo was infused in healthy, nondiabetic young men (*n* = 10) during a 4-hour basal period followed by an intravenous glucose tolerance test (IVGTT). TNF-**α** lowered insulin levels by 12% during the basal period (*P* < 0.05). In response to the IVGTT, insulin levels increased markedly in both trials, but there was no difference between trials. Compared to placebo, TNF-**α** did not affect EGP during the basal period. Our results indicate that TNF-**α** acutely lowers basal plasma insulin levels but does not impair GSIS. The mechanisms behind this are unknown but we suggest that it may be due to TNF-**α** increasing clearance of insulin from plasma without impairing beta-cell function or hepatic insulin sensitivity.

## 1. Introduction

Type 2 diabetes is caused by a progressive decrease of insulin sensitivity and pancreatic *β*-cell function. Insulin resistance is characterized by decreased sensitivity to insulin not only in skeletal muscle but in all insulin-sensitive tissue which includes adipose tissue and the liver [1]. Although the mechanisms behind insulin resistance are not completely clear, low-level inflammation seems to play a role in the development of type 2 diabetes and insulin resistance [2]. Animal models as well as human studies have shown that obese and diabetics have increased production of proinflammatory cytokines—such as tumor necrosis factor-*α* (TNF-*α*)—in adipose tissue [3]. It has been shown that TNF-*α* can induce insulin resistance in humans via serine phosphorylation of the insulin receptor and via inhibition of the Akt substrate 160 phosphorylation [4, 5]. TNF-*α* may also have a role in impaired *β*-cell function and decreased glucose-stimulated insulin secretion. It has been shown *in vitro* that TNF-*α* can decrease the glucose induced insulin release from pancreatic *β*-cells through activation of nuclear factor kappaB (NF-*κ*B) and local production of nitrous oxide [6, 7]. Chen et al. showed that decreased TNF-*α* levels after transient insulin therapy correlates with improved *β*-cell function in type 2 diabetics, but the question remains whether this improvement was due to the reduced TNF-*α* level or the insulin therapy [8]. Treatment with anti-TNF-*α* in humans has so far not shown any results indicating a beneficial effect on insulin resistance or GSIS [9, 10]. Thus, the effect of TNF-*α* on GSIS *in vivo* in humans remains to be established.

Our hypothesis was that a low-dose TNF-*α* infusion would decrease both basal (BIS) and glucose-stimulated insulin secretion (GSIS). We therefore decided to investigate whether TNF-*α* would have an acute effect on BIS as well as the insulin response to an intravenous glucose tolerance test (IVGTT).

## 2. Materials and Methods

The overall study design has been described earlier [11, 12]. Ten healthy males participated in the study. The subject data is shown in Table 1. The subjects first underwent a clinical examination and standard blood samples were drawn to ensure that the subjects did not suffer from any infections or other diseases. Exclusion criteria were obesity (BMI > 25), diabetes, and chronic disease. The subjects received both oral and written information about the risks and discomforts of the trial and signed a written consent. The study was approved by the Ethics Committee of Copenhagen and Frederiksberg (file number KF 01-215/04) in accordance with the Helsinki Declaration.

### 2.1. Experimental Design

The subjects had been instructed not to eat or drink anything except water at least 10 hours prior to the beginning of the experiment. Using a randomized and double-blinded design, each subject underwent two separate trials, consisting of a 5.5-hour infusion of either saline or recombinant human TNF-*α* (TNF, Beromun, Boeringer-Ingelheim, Denmark) through an antecubital vein catheter. The TNF-*α* was administered in saline with 20% human albumin at a rate of  700 ng·h^−1^·m^−2^. Placebo consisted of saline with 20% human albumin. A catheter (Arrow, PA, USA) for blood sampling was placed in the femoral artery (20-gauge). After local anesthesia, the artery was cannulated below the inguinal ligament and the catheter was advanced ~10 cm proximally using guidewire (Seldinger) technique [13]. During the trial, arterial blood samples were drawn into EDTA tubes every 30 min, separated into plasma, and stored at −80°C until analysis.

### 2.2. IVGTT

In both trials an IVGTT was performed. After 4 hours of TNF-*α* infusion, a 0.3 g·kg^−1^ bolus of 20% glucose monohydrate (C_6_H_12_O_6_·H_2_O, SAD, Denmark) was injected into the veins within 30 seconds. Arterial blood samples for glucose and insulin measurements were collected after 1, 2, 3, 4, 5, 7, 9, 11, 13, 15, 20, 25, 30, 40, 50, 60, 70, 80, and 90 minutes. The blood was separated into plasma and stored at −80°C until analysis.

### 2.3. Plasma Concentrations

IL-6, TNF-*α*, and insulin were measured using commercially available ELISA kits (IL-6: #HS600B, R&D Systems, Minneapolis, MN, USA; TNF-*α*: #HSTA00C, R&D Systems; and insulin: #K6219, DakoCytomation, St. Charles, MO, USA). Detection limits were 0.1 pg/mL for IL-6, 0.3 pg/mL for TNF-*α*, and 10 pmol/L for insulin. Mean intra- and interassay coefficients of variance (CV) were 4% and 6% for insulin, 3% and 20% for TNF-*α*, and 6% and 20% for IL-6. Free fatty acids (FFA) were measured as previously described [14]. Glucose measurements were made with an ABX Hexokinase Assay (#A11A01667, ABX Diagnostics, Montpellier, France) on a Cobas Fara robot (Roche) [15]. Leukocytes were counted using the Sysmex XE-2100 (Sysmex Corporation, Kobe, Japan) equipment at the Department of Clinical Biochemistry 3011 at Rigshospitalet, Copenhagen.

### 2.4. Stable Isotopes

[6,6-^2^H_2_]-glucose was measured as previously described [11].

Glucose rate of appearance (*R*_*a*_) was calculated using a single-pool, steady-state model, where *R*_*a*_ = tracer inflow/*E*_*p*_ (Tracer inflow is the glucose tracer infusion rate expressed as *μ*mol/min·kg and *E*_*p*_ is the tracer-to-tracee ratio) [16].

### 2.5. Statistics

The distribution of all variables was evaluated using histograms and probability plots. In case of nonnormal distribution, data was log_10_-transformed in order to obtain normal distribution. The following variables were log_10_-transformed: lymphocytes, monocytes, neutrocytes, and levels of plasma TNF-*α*, IL-6, glucose, and insulin. Accordingly, data are presented as means or geometric means with 95% confidence intervals. The overall effect of time and treatment was analyzed using a 2-way repeated measures mixed-model analysis (SAS release 9.1, SAS Institute Inc., Cary, NC, USA). Differences over time in each separate group were evaluated using a 1-way repeated measures mixed-model analysis. In case significant main effects were found, post-hoc analysis was performed by calculation of false discovery rates [17]. A Student's *t*-test was used to assess differences between TNF-*α* and placebo treatment when only 2 points were compared. The level of significance was set at *P* < 0.05.

## 3. Results

### 3.1. Inflammatory Response

Both plasma TNF-*α* and IL-6 concentrations rose in response to the TNF-*α* infusion (Table 2). Plasma TNF-*α* concentration peaked after 2 hours with a 20-fold increase compared to controls (*P* < 0.05). Plasma IL-6 concentration increased up to 6-fold with a peak after 3 hours compared to controls (*P* < 0.05). Both TNF-*α* and IL-6 concentrations decreased in response to the IVGTT compared to pre-IVGTT levels (240 versus 330 min: *P* < 0.05 in both trials).

Compared to placebo, the number of monocytes and lymphocytes (Table 2) decreased in response to the TNF-*α* infusion (*P* < 0.05), whereas the number of neutrocytes increased (*P* < 0.05). In addition, TNF-*α* infusion was associated with small increases of the tympanic temperature and the heart rate (*P* < 0.05). Otherwise the subjects showed no symptoms of the TNF-*α* infusion, and accordingly, the subjects were unable to distinguish the placebo trial from the TNF-*α* trial.

### 3.2. Endogenous Glucose Production

**Figure 1:**
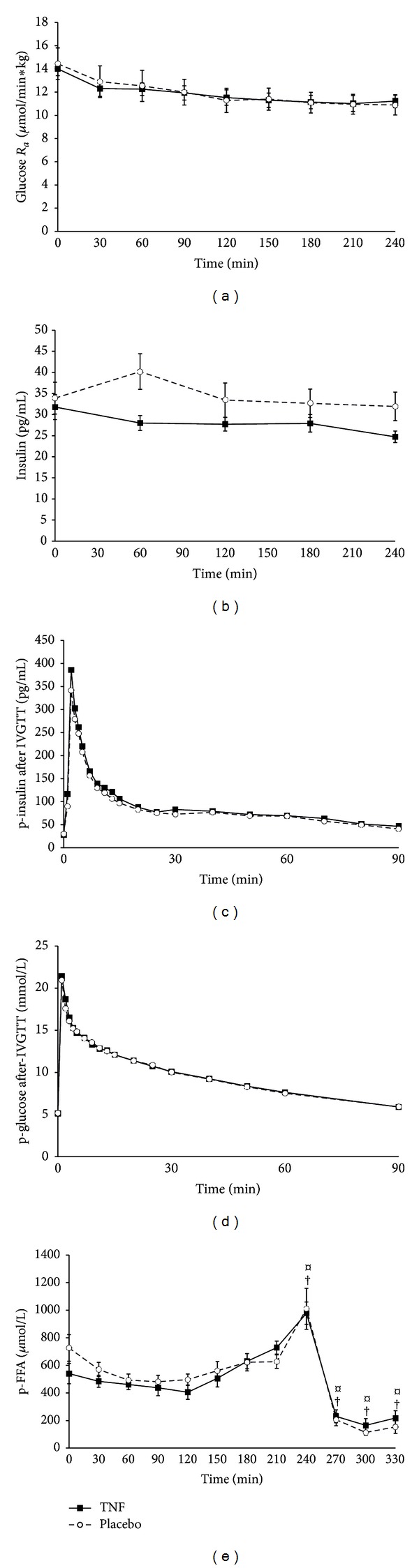
(a) Glucose rate of appearance (*R*_*a*_). Continuous infusion of recombinant TNF-*α* (700 ng·h^−1^·m^−2^) started at 0 min. Shown are geometric means with 95% CI. There was no difference between the two groups. (b) Plasma insulin during infusion of recombinant TNF-*α*. Shown are geometric means with 95% CI. During the basal period, plasma insulin concentration decreased over time in the TNF-*α* trial but not in the placebo trial (treatment × time, *P* = 0.02). (c) Plasma insulin during IVGTT. Shown are geometric means with 95% CI. There was no difference between the two groups. (d) Plasma glucose after IVGTT. Shown are geometric means with 95% CI. There was no difference between the two groups. (e) Free fatty acid (FFA) concentration before and after IVGTT. *¤*, different (*P* < 0.05) from time = 0 min in placebo trial; †, different from time = 0 min in TNF-*α* trial. Shown are geometric means with 95% CI. There was no difference between the two groups.

Glucose *R*_*a*_ decreased over time from 14.0 (12.7–15.3) to 11.2 (10.2–12.3) *μ*mol·min^−1^·kg^−1^ in the TNF-*α* trial (*P* < 0.05) and from 14.4 (11.8–17.1) to 10.9 (9.3–12.4) *μ*mol·min^−1^·kg^−1^ in the placebo trial (*P* < 0.05), respectively. There was no difference (*P* > 0.05) between the TNF-*α* and the control group (Figure 1(a)).

### 3.3. Plasma Insulin and Glucose

During the basal period, plasma insulin concentration decreased over time in the TNF-*α* trial but not in the placebo trial (Figure 1(b), treatment × time, *P* = 0.02). Post hoc analysis revealed a significant difference between groups at times = 60 mins and 240 mins (Student's *t*-test, *P* < 0.05), while there was no difference between groups at time = 0. Following the injection of the glucose bolus, plasma insulin peaked after 2 minutes (Figure 1(c)), however, with no difference (*P* > 0.05) between placebo and TNF-*α* trials (350 (240–460) pg/mL and 297 (109–389) pg/mL in the TNF-*α* trial and placebo trials, resp.).

Basal period glucose levels were not different (*P* > 0.05) in the groups. Following the IVGTT, plasma glucose increased 4-fold to reach a mean peak concentration of 21.4 (20.3–22.6) mmol/L in the TNF-*α* trial and 21.0 (20.0–22.0) mmol/L in the placebo trial. Subsequently, the glucose concentration decreased to reach basal levels after 90 minutes. Again, there was no difference (*P* > 0.05) in the glucose concentrations between the TNF-*α* and the placebo trials (Figure 1(d)).

### 3.4. Free Fatty Acids

Plasma FFA concentration showed a steady rise from 2 hours until the end of the basal period (Figure 1(e)). FFA levels increased in both groups up to 1.8-fold in the TNF-*α* trial and 1.4-fold in the placebo trial (*P* < 0.05). Following IVGTT, FFA levels decreased in both groups to reach values 6-fold (TNF-*α*, *P* < 0.05) and 9-fold (placebo, *P* < 0.05) lower than the pre-IVGTT values. There was no effect of TNF-*α* on FFA levels neither during the basal period nor after-IVGTT.

## 4. Discussion

The main purpose of this study was to examine whether acute low-level increase of TNF-*α* had an effect on the basal and glucose-stimulated insulin secretion. We found an indication that TNF-*α* lowered the basal insulin levels but did not affect the glucose-stimulated insulin secretion.

Cell culture studies have not provided consistent results regarding the effect of TNF-*α* on pancreatic *β*-cells. Sternesjo et al. as well as Bouzakri et al. did not find a detrimental effect of TNF-*α* on *β*-cells or basal insulin secretion *in vitro* [18, 19]. Other studies, however, have demonstrated a detrimental effect of TNF-*α* on the glucose-stimulated insulin secretion [6, 7]. In human trials there is some evidence supporting an important role of circulating TNF-*α* on *β*-cell function. In two never studies, treatment with TNF-*α* inhibitors showed an improvement in blood glucose and a lower risk of diabetes [20, 21]. Other studies, however, have failed to show this effect. Two studies showed that administration of the TNF-*α* receptor antagonist etanercept did not seem to have any effect on the glucose-stimulated insulin response or reduced insulin resistance [10, 22], while treatment with infliximab, an anti-TNF-*α* antibody, may improve insulin sensitivity in patients with rheumatoid arthritis [23, 24]. It has also been proposed that TNF-*α* per se does not reduce GSIS but rather may act by potentiating the effect of FFA and other proinflammatory cytokines such as IFN-*γ*, IL-6, and IL-1*β* [25, 26]. In this study, there was a rise in IL-6 following the TNF-*α* infusion, but still we did not observe an effect of TNF-*α* on GSIS. Therefore, our data does not support this hypothesis. Our results support previous human studies suggesting that TNF-*α* plays a role in development of type 2 diabetes mainly by inducing insulin resistance and not by impairment of the insulin secretion by the *β*-cell [5, 27].

The concentration of plasma TNF-*α* in this study was slightly higher than in obese and diabetics but much lower than in patients with sepsis. TNF-*α* concentrations employed *in vitro* are often much higher than the plasma concentrations found in human patients with obesity and type 2 diabetes, and therefore it may be difficult to extrapolate from findings obtained *in vitro* to *in vivo* [6, 28]. It is possible that higher doses of TNF-*α* would have impaired the glucose-stimulated insulin response during the IVGTT, but, based on the present data, it does not seem likely that low levels of circulating TNF-*α* have any major effect on the insulin response to glucose. Also, it is possible that a chronic elevation of plasma TNF-*α*—as observed in, for example, obesity—might affect *β*-cell function over longer time. However, based on our results, the possible effect of circulating TNF-*α* on *β*-cell function appears to be less important.

We found an acute reduction in the levels of IL-6 and TNF-*α* 90 minutes after-IVGTT. This is surprising since other studies have found increases in inflammatory markers after glucose challenges [29–31]. These studies, however, were based on oral glucose testing and the time frame for measuring was much longer than ours. Indeed, the effect of glucose testing on inflammatory markers may not present itself until several hours after the challenges. This would be in accordance with the findings of Wopereis et al., where the rise in inflammatory markers does not happen until several hours after the glucose challenge, if at all [32].

The study was also designed to examine whether TNF-*α* infusion had an effect on endogenous glucose production (EGP) during fasting. This has previously only been examined during hyperinsulinemic clamps, where TNF-*α* had no effect on EGP [5]. The high levels of insulin may have concealed the effect of TNF-*α* and we therefore wanted to investigate whether TNF-*α* would increase EGP when fasting levels of insulin were present. We found that TNF-*α* did not affect EGP. This is in accordance with a previous study by Plomgaard et al. [5] that did not show any effect of TNF-*α* on EGP in healthy young subjects during an euglycemic hyperinsulinemic clamp. In summary, TNF-*α* seems to act mainly on peripheral insulin action and insulin clearance rather than directly on the insulin secretion by pancreatic *β*-cells.

The decreased basal insulin levels induced by the TNF-*α* infusion could be due to either (1) impaired insulin secretion, (2) increased disposal of insulin in skeletal muscle and adipose tissue, or (3) via increased clearance of insulin by the liver or kidneys [33]. Which of the mechanisms that are responsible remains to be investigated. However, since the glucose-stimulated insulin response was equally high during infusion with TNF-*α* and placebo, impaired *β*-cell function does not seem likely nor does it seem likely that the lower plasma insulin levels during TNF-*α* are due to increased peripheral disposal of insulin, since peripheral insulin signaling is acutely impaired by low-level infusion of TNF-*α* [5].

In conclusion, we have showed that acute TNF-*α* infusion lowers the basal insulin levels but does not affect the insulin response to glucose. To investigate which exact mechanisms are responsible for decreased basal insulin levels, further studies are needed.

## Figures and Tables

**Table 1:** Subject characteristics.

Subjects (*n*)	10
Age (yrs)	26.0 ± 4.2
Weight (kg)	73.9 ± 6.9
Height (cm)	181 ± 5
BMI (kg/m^2^)	22.6 ± 1.9
Fasting plasma glucose (mmol/L)	4.5 ± 0.7
Fasting plasma insulin (pg/mL)	29.8 ± 4.0

Values are means ± S.E.M. BMI: body mass index.

**Table 2:** Subject measurements.

Variable (unit)	Time (min)	Trial	
		Placebo	TNF-*α*
TNF-*α* (pg/mL)	0	0.8 ± 0.1	0.8 ± 0.1
	240	0.8 ± 0.1	15.9 ± 1.7^§†^
	330	0.9 ± 0.1	14.2 ± 1.1^§†^

IL-6 (pg/mL)	0	1.1 ± 0.2	1.0 ± 0.2
	240	1.5 ± 0.2	9.2 ± 1.0^§†^
	330	1.9 ± 0.1	7.0 ± 0.7^§†^

Tympanic temperature (°C)	0	36.3 ± 0.1	36.3 ± 0.2
	240	36.5 ± 0.1	37.0 ± 0.1^§†^
	330	36.4 ± 0.2	37.2 ± 0.2^§†^

HR (beats/min)	0	56 ± 3.5	60 ± 2.7
	240	58 ± 3.3	70 ± 2.1^§†^
	330	60 ± 2.6	77 ± 2.6^§†^

MAP (mmHg)	0	87 ± 1.6	87 ± 3.3
	240	83 ± 1.4	84 ± 1.8
	330	88 ± 2.7	85 ± 2.8

Lymphocytes (10^9^ cells/L)	0	1.8 ± 0.1	1.9 ± 0.1
	240	2.0 ± 0.2	1.1 ± 0.1^§†^
	330	1.7 ± 0.1	0.9 ± 0.1^§†^

Neutrocytes (10^9^ cells/L)	0	3.3 ± 0.4	3.3 ± 0.5
	240	3.7 ± 0.4	7.1 ± 0.5^§†^
	330	3.6 ± 0.3	5.8 ± 0.4^§†^

Monocytes (10^9^ cells/L)	0	0.47 ± 0.03	0.44 ± 0.04
	240	0.47 ± 0.04	0.32 ± 0.04^§†^
	330	0.50 ± 0.04	0.30 ± 0.04^§†^

Values are means ± S.E.M. MAP: mean arterial pressure; HR: heart rate.

^§  ^Difference (*P* < 0.05) between TNF-*α* and placebo trial; ^†^difference (*P* < 0.05) from time = 0 min.
